# The complete mitochondrial genome of the Australian ghost bat *Macroderma gigas*

**DOI:** 10.1080/23802359.2021.1962754

**Published:** 2021-08-13

**Authors:** Jaco D. Zandberg, Wayne G. Reeve, Peter B. S. Spencer

**Affiliations:** a Medical, Molecular and Forensic Sciences, Murdoch University, Murdoch, Australia; b Environmental and Conservation Sciences, Murdoch University, Murdoch, Australia

**Keywords:** Chordata, ghost bat, mitogenome, chiroptera, Megadermatidae

## Abstract

The Ghost bat *Macroderma gigas* is a monotypic bat species that is endemic to northern Australia and named on the basis of the large size of its partially conjoined ears. It is the only carnivorous bat found in Australia and its conservation status is currently listed as Vulnerable. Here, we describe the complete mitochondrial genome of *M. gigas* and compare it to other vertebrates. The *M. gigas* circularized mitogenome was 16,661 bp and contained 13 protein-coding genes, two rRNA genes, 22 tRNAs and a control region (D-loop) of 1228 bp. Phylogenetic analysis of available entire mitogenomes reveals that *Macroderma gigas* is most closely related to the Indian false vampire bat *Megaderma lyra* in the family Megadermatidae (false vampire bats).

The Ghost bat *Macroderma gigas* is the only flying carnivorous bat that is endemic to Australia. It has partially conjoined ears and is the largest bat in the family Megadermatidae (false vampires; Hudson and Wilson 1986). The name Ghost bat is derived from the predominant color of its fur which may be near white to pale gray (Hudson and Wilson 1986). The Ghost bat has comparatively large and well-developed eyes for nocturnal vision and has large ears and prominent nasal appendage which provides the means to manipulate, direct and receive echolocation signals to detect prey. Since its discovery in 1880, the habitat range has significantly contracted and it is now listed as Vulnerable in the Red List (IUCN). It is estimated that only several thousand ghost bats remain in existence today (Hudson and Wilson 1986) , concentrated in three population centers; in Western Australia in the Northern Pilbara and Kimberley regions, and in Queensland (Hoyle et al. 2001). Female bats congregate in distinct maternal sites and stay in these congregations until young are reared. Females across maternal populations have expressed alleles that are distinguishable which implies that separation of populations has occurred through evolutionary time (Wilmer et al. 1999).

Both nuclear (Ottewell et al. 2020) and mtDNA genomes can make a substantial contribution to phylogenetics and, to our knowledge, the mitochondrial genome has not been established for any species of *Macroderma*. In this study, we have therefore determined the complete mitochondrial genome for *Macroderma gigas*. DNA was sourced from voucher material from a captive individual located in Perth Zoo, (–31.976 S; 115.852 E; lab number 16-879).

The DNA was sequenced using the Illumina MiSeq Platform (Illumina, San Diego, CA) which produced 1,496,976 paired-reads reads totaling 0.449 Gbp. The 300 bp paired-end reads were trimmed by BBDuck at Q13 and then merged using the Geneious Prime v2019.2.1 (Biomatters^®^, NZ) internal merging tool. We then *de novo* assembled the complete mitogenome using 4849 reads (0.73% of the total trimmed and merged paired end reads) to produce a circular mitogenome of size 16,661 bp with 87x coverage. Annotations for the protein-coding, tRNA, and rRNA genes for the finished mitogenome were retrieved from other finished mitogenomes using the ‘Annotate and Predict’ feature of Geneious Prime v2019.2.1 (Biomatters^®^, NZ). The sequence with annotated features has been deposited in GenBank under the Accession Number MW006543. The *M. gigas* complete mitogenome sequence length was established to be 16,661 bp with a typical vertebrate mitogenome organization (Nilsson et al. 2004) containing 13 protein-coding genes, two rRNA genes, 22 tRNA genes, and a noncoding control region (D-loop). The overall base composition was 28.6% A, 23.2% T, 32.1% C, and 16.1% G, with a GC% content of 48.2% which is dissimilar to the other bats compared. Of the 13 protein-coding genes, eight initiated with ATG, while three started with ATA (CYTB, ND2 and ND3) and two with GTG (ND5 and ND6). Eight protein-coding genes ended with TAA; five of which had TAA as the stop codon in the gene sequence (ATP6, COX2, ND4L, ND5 and ND6) and the other three had a stop codon that would be produced from the addition of 3′ A-residues to the mRNA (COX1, COX3 and ND4). Another four ended with TAG (ATP8, ND1, ND2 and ND3), one with AGA (CYTB).

Phylogenetic analysis was conducted using entire mitogenome sequences (including the control region) of 46 finished mitogenomes (Figure 1), using the GTR + G + I model of best fit in MEGA-X (Nei and Kumar 2000). The evolutionary history was inferred by using the maximum-likelihood method and general time reversible model (Nei and Kumar 2000), which provided the lowest Bayesian Inference Criterion (BIC) score. The bootstrap consensus was inferred from 1000 replicates (Felsenstein 1985). The tree with the highest log likelihood (-419230.44) is shown. The percentage of trees in which the associated taxa clustered together is shown next to the branches. Initial tree(s) for the heuristic search were obtained automatically by applying Neighbor-Join and BioNJ algorithms to a matrix of pairwise distances estimated using the Maximum Composite Likelihood (MCL) approach, and then selecting the topology with superior log likelihood value. A discrete Gamma distribution was used to model evolutionary rate differences among sites (5 categories (+G, parameter = 0.7882)). The rate variation model allowed for some sites to be evolutionarily invariable ([+I], 17.92% sites). The tree is drawn to scale, with branch lengths measured in the number of substitutions per site. There were 21,014 positions in the final dataset. Evolutionary analyses were conducted in MEGA X (Kumar et al. 2018). The phylogenetic comparison placed *M. gigas* in the Megadermatidae family order, with its closest relative being the Indian false vampire bat (*Megaderma lyra*).

**Figure 1.**
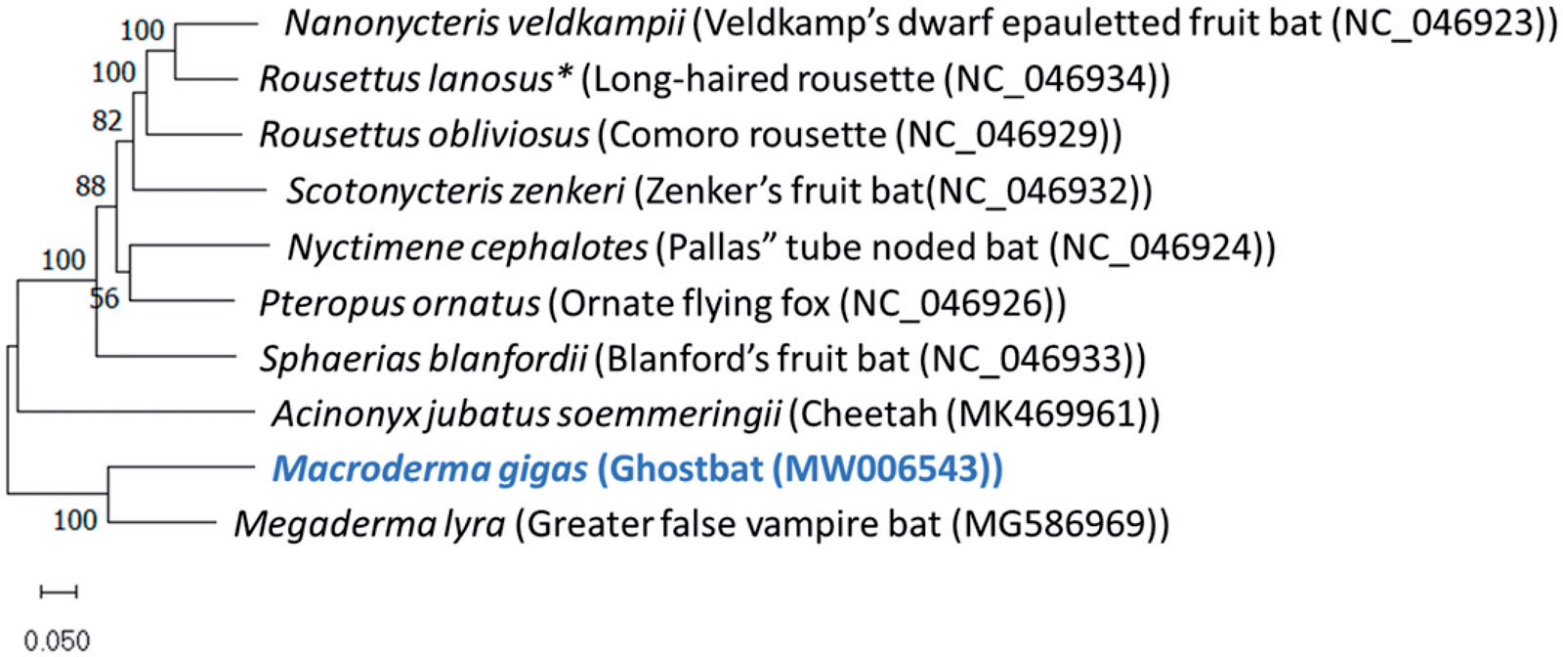
Phylogenetic placement of *Macroderma gigas* based on a truncated comparison of the rRNA and coding DNA sequences to other entire vertebrate mitogenomes. *Also known as *Stenonycteris lanosus*.

## Geolocation information

Geospatial coordinates from voucher material for the Ghost bat *Macroderma gigas* was sourced from a captive individual located in Perth Zoo, (-31.976 S; 115.852 E; lab number 16-879).

## Data Availability

The data that support the findings of this study are available from either GenBank (see Figure 1) or from the corresponding author, [PS], upon reasonable request. The complete mitochondrial sequence has been deposited in the NCBI online database under the accession MW006543 (https://www.ncbi.nlm.nih.gov/nuccore/MW006543.1/). Sequencing data can be retrieved from NCBI Biosample (SAMN16048702) SRA platform (SRX11310596) (https://www.ncbi.nlm.nih.gov/sra?LinkName=biosample_sra&from_uid=16048702)
